# Supplementary material for: Anti-inflammatory effects of neutral lipids, glycolipids, phospholipids from Halocynthia aurantium tunic by suppressing the activation of NF-κB and MAPKs in LPS-stimulated RAW264.7 macrophages
Source: PLoS One. 2022 Aug 15;17(8):e0270794. doi: 10.1371/journal.pone.0270794 (PMC9377571; doi:10.1371/journal.pone.0270794)

Original western blot gel image data

1. Neutral Lipids

<u>Replication 1</u>

- **p-NF-κB-p65**

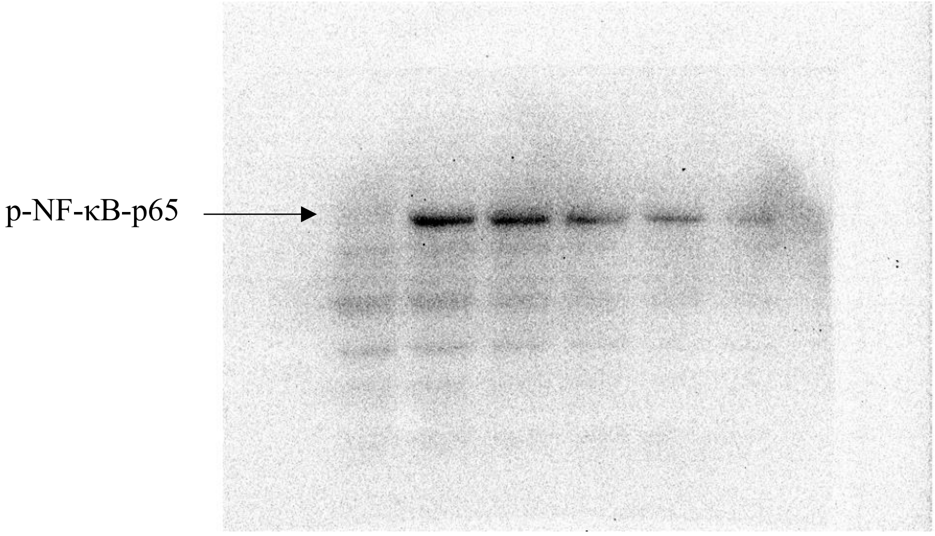

- **p-p38**

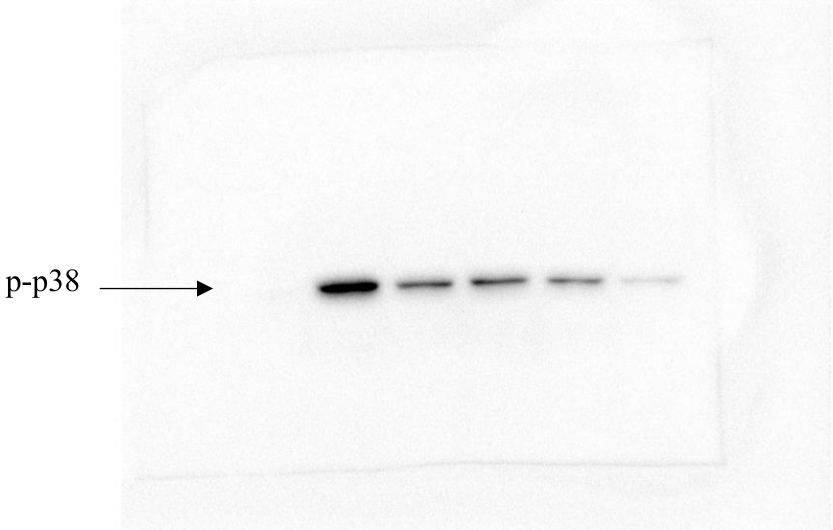

- **p-JNK**

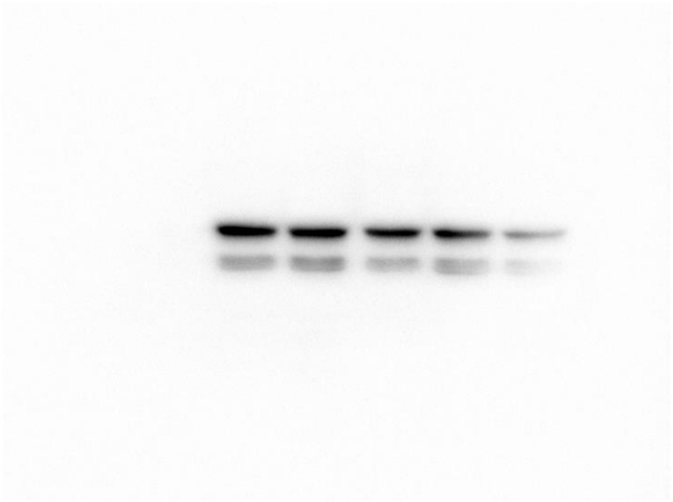

- **p-ERK 1/2**

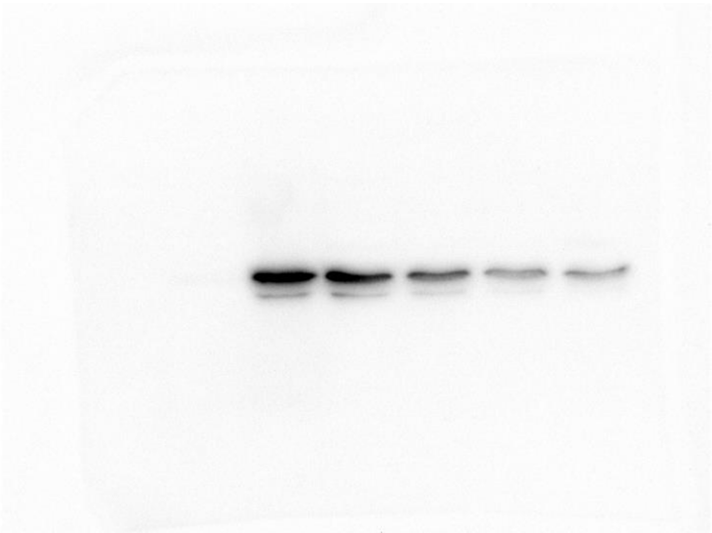

- **α-Tubulin**

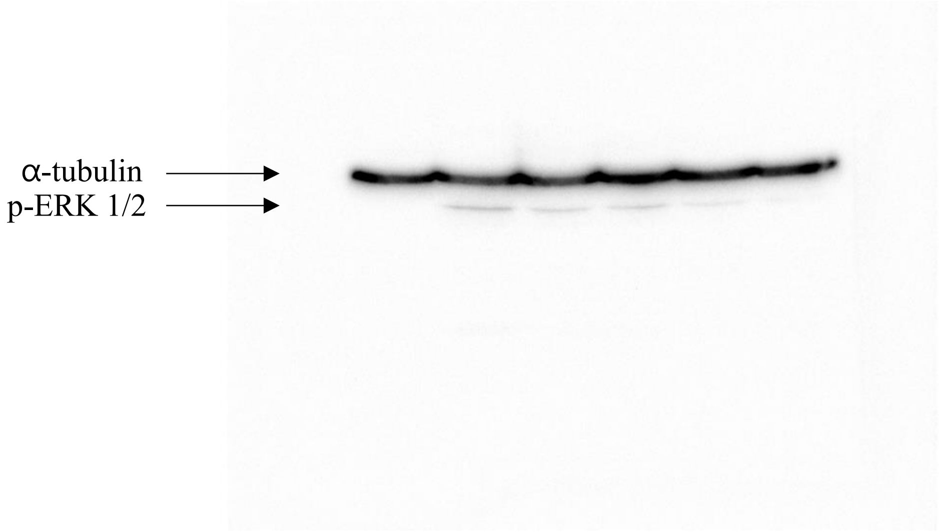

Replication 2

- **p-NF-κB-p65**

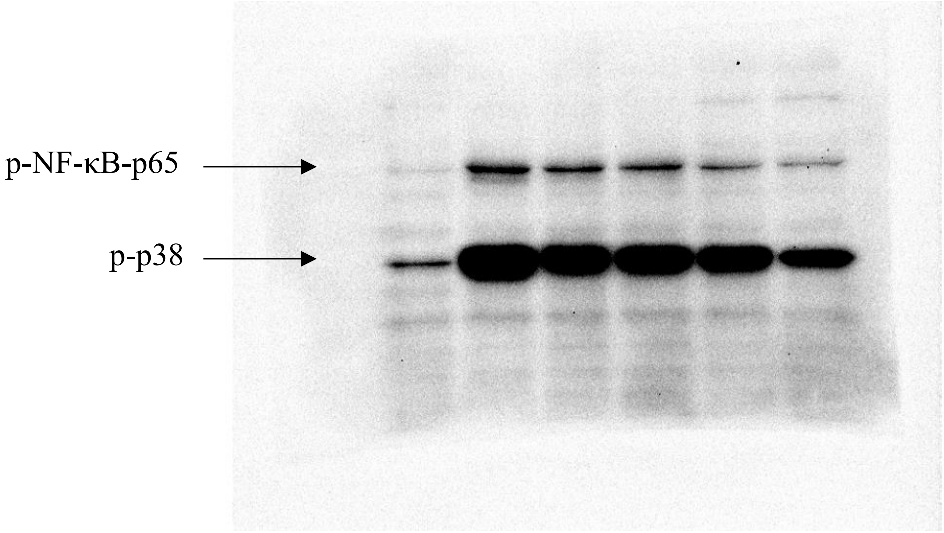

- **p-p38**

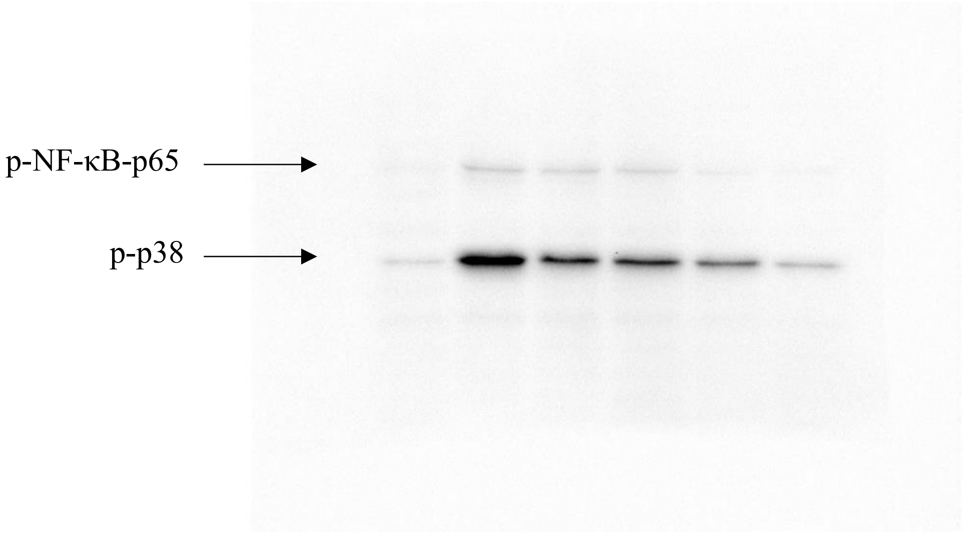

- **p-JNK**

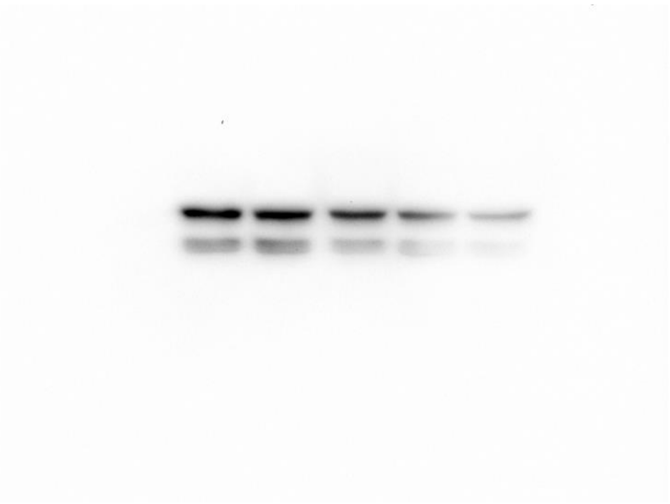

- **p-ERK 1/2**

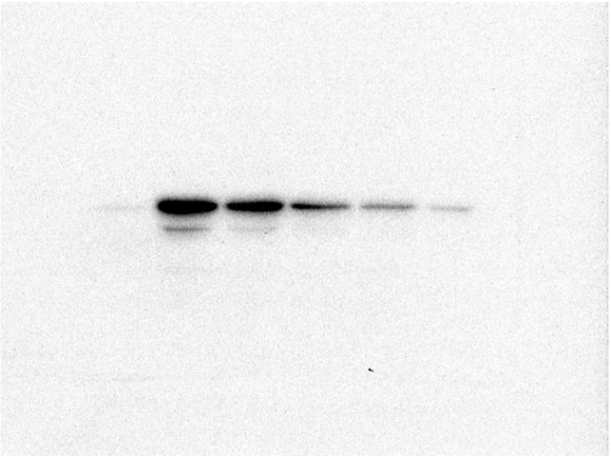

- **α-Tubulin**

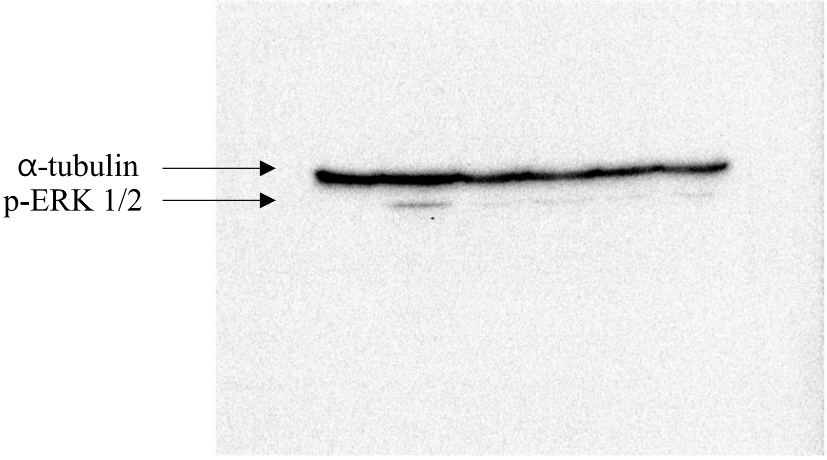

<u>Replication 3</u>

- **p-NF-κB-p65**

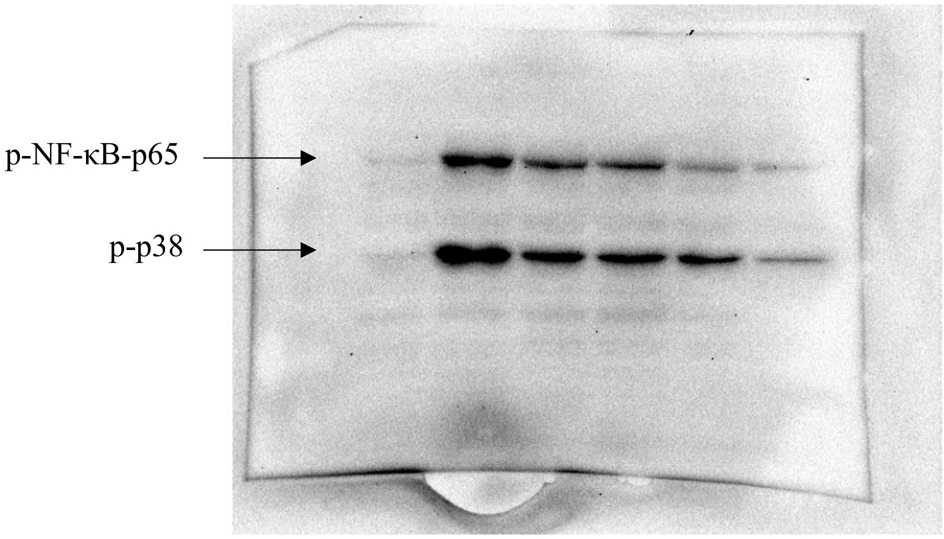

- **p-p38**

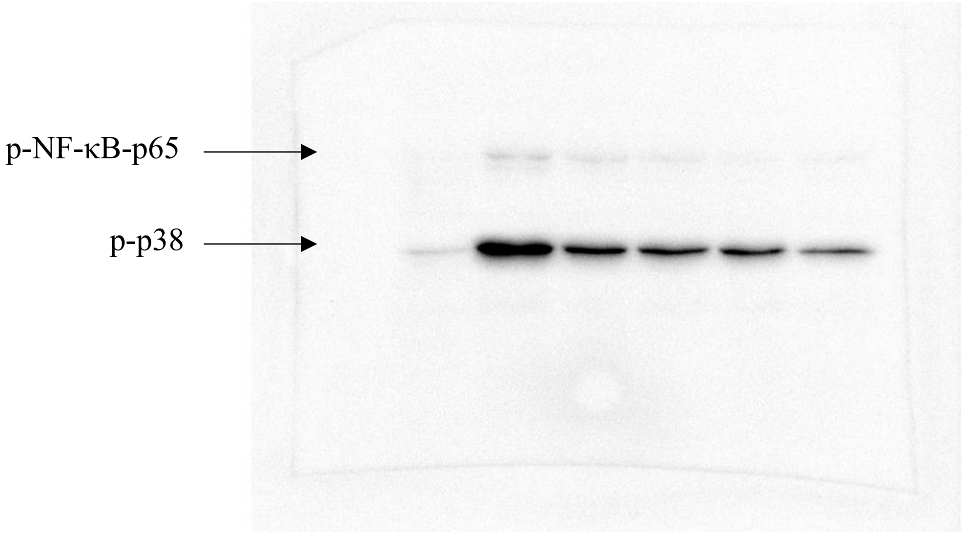

- **p-JNK**

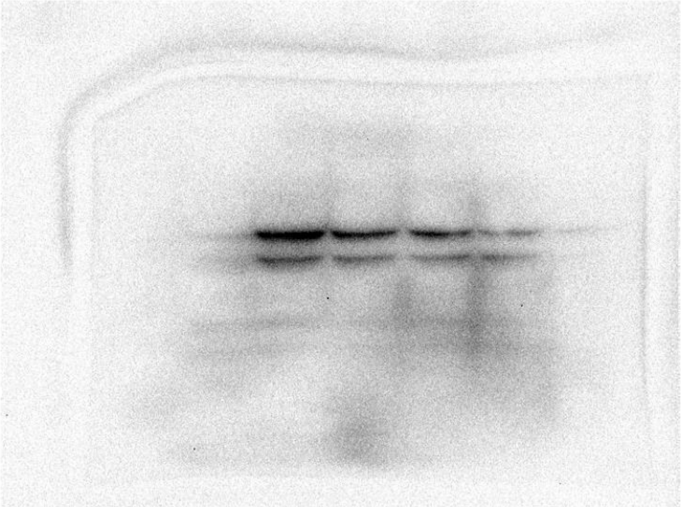

- **p-ERK 1/2**

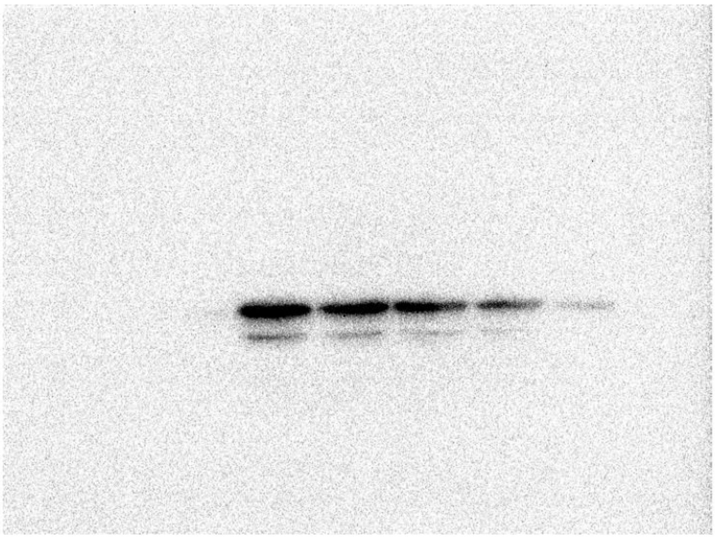

- **α-Tubulin**

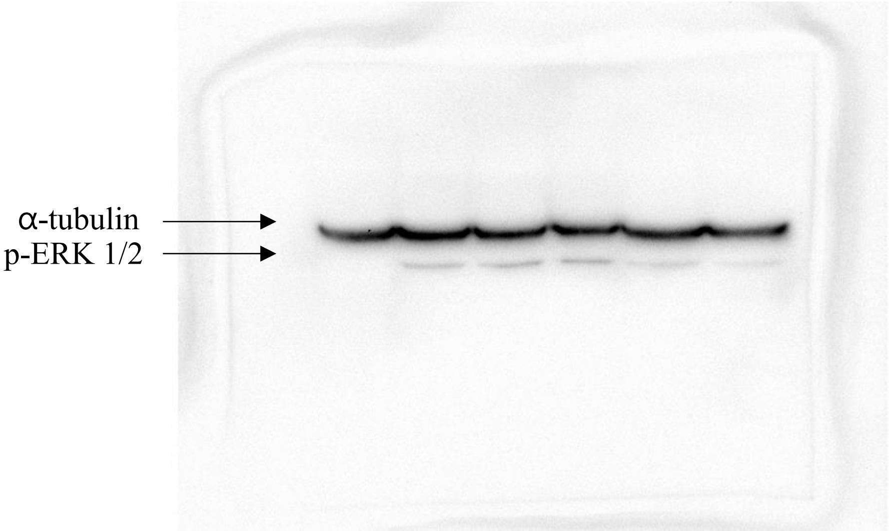

2. Glycolipids

<u>Replication 1</u>

- **p-NF-κB-p65**

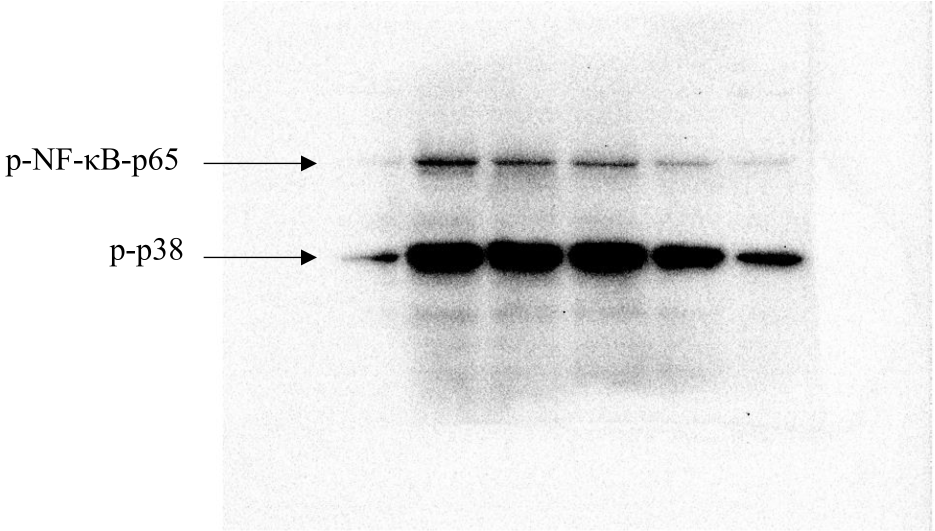

- **p-p38**

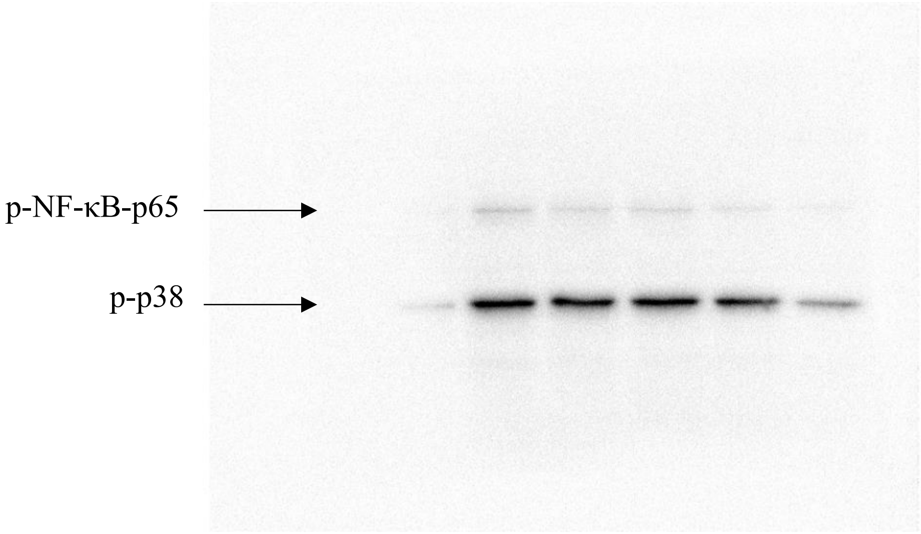

- **p-JNK**

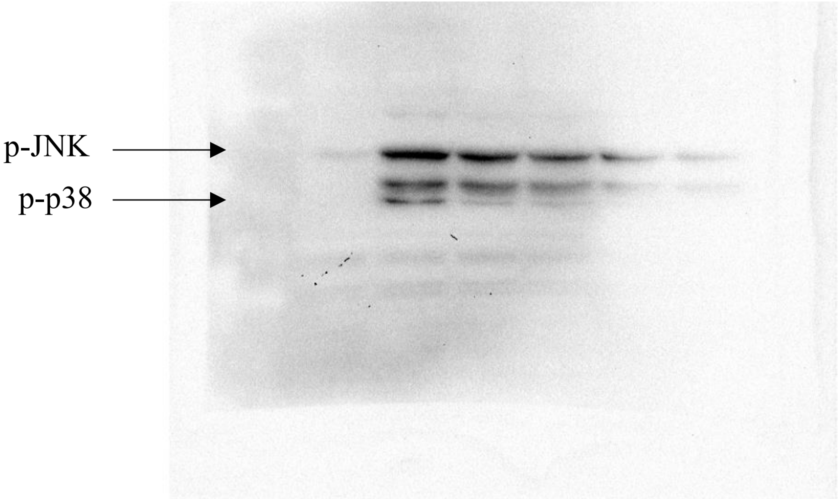

- **p-ERK 1/2 and α-Tubulin**

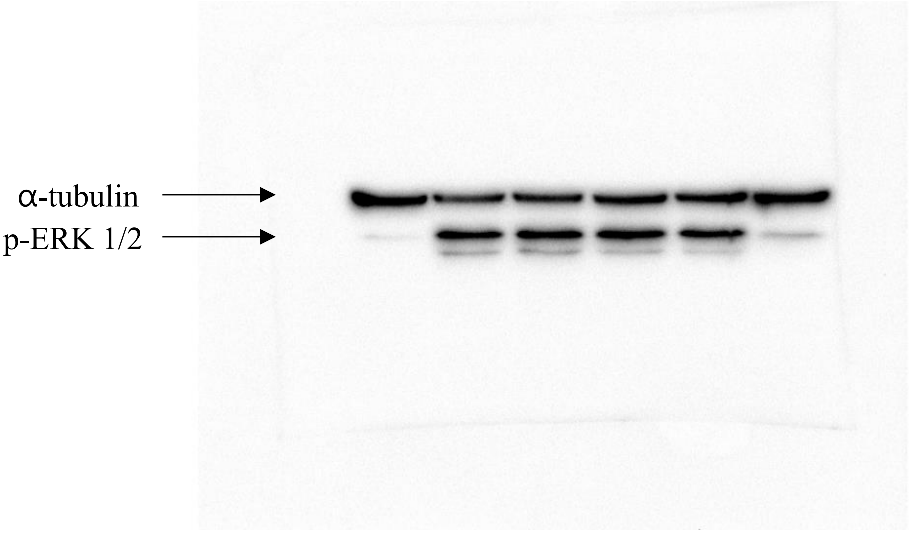

Replication 2

- **p-NF-κB-p65**

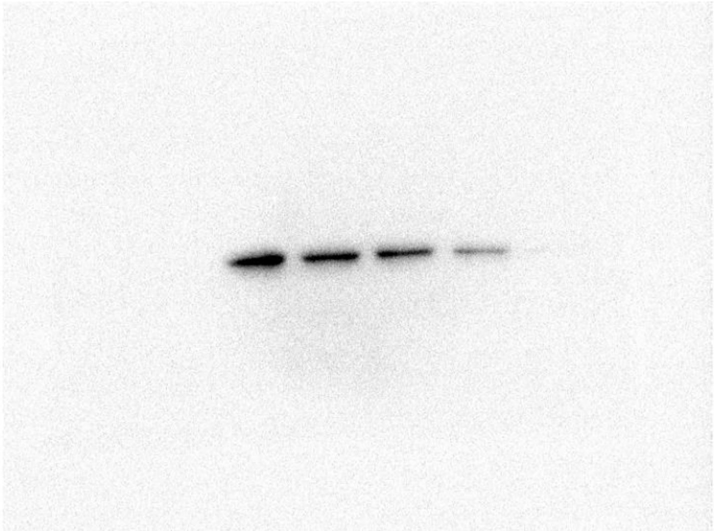

- **p-p38**

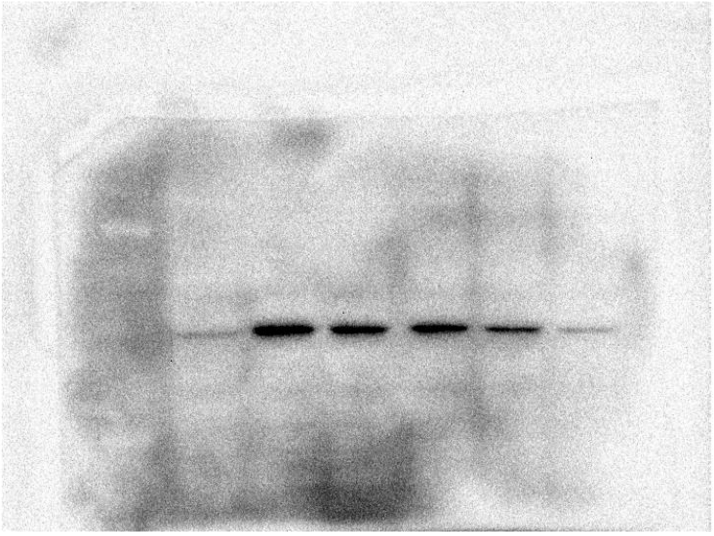

- **p-JNK**

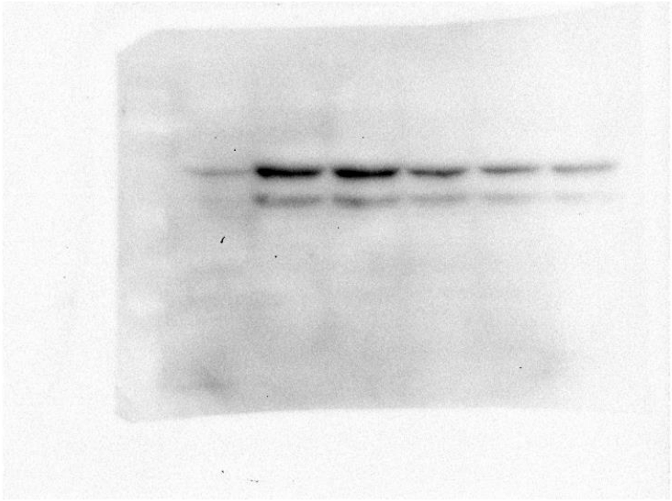

- **p-ERK 1/2 and α-Tubulin**

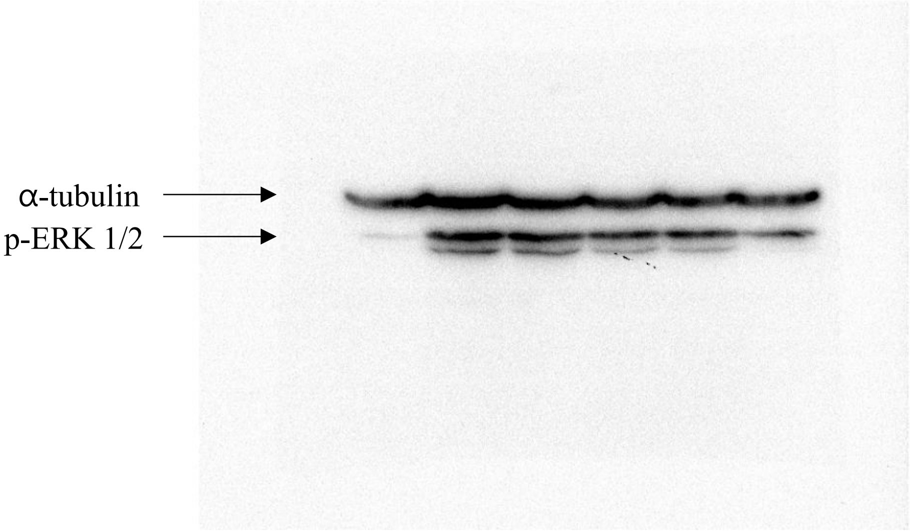

<u>Replication 3</u>

- **p-NF-κB-p65**

p-NF-κB-p65

p-p38

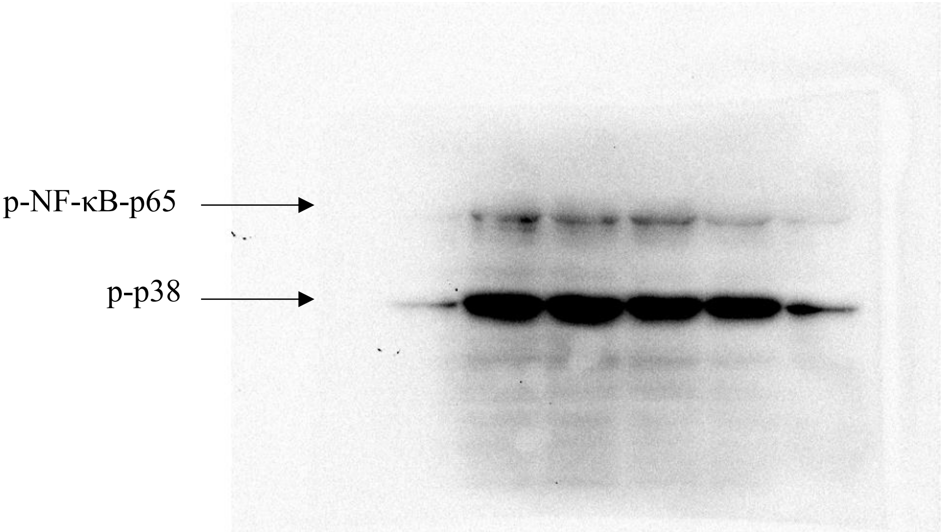

- **p-p38**

p-NF-κB-p65

p-p38

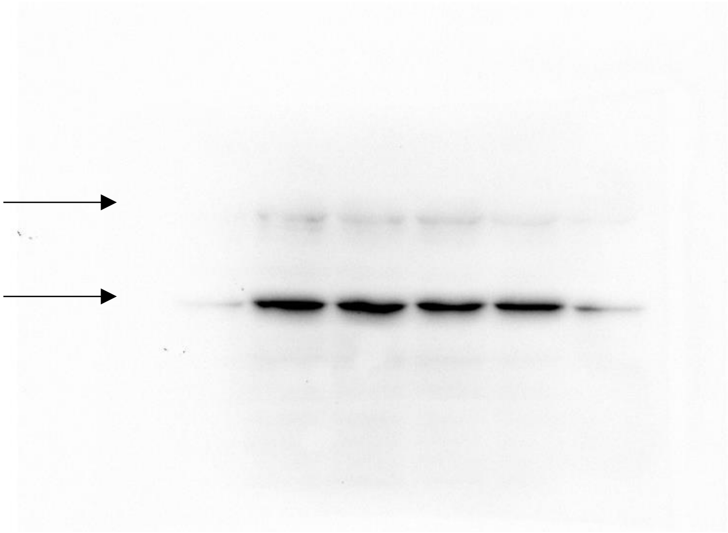

- **p-JNK**

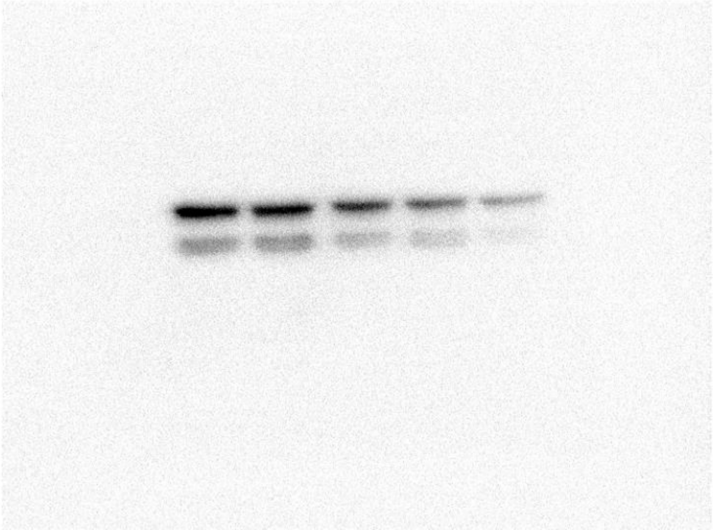

- **p-ERK 1/2**

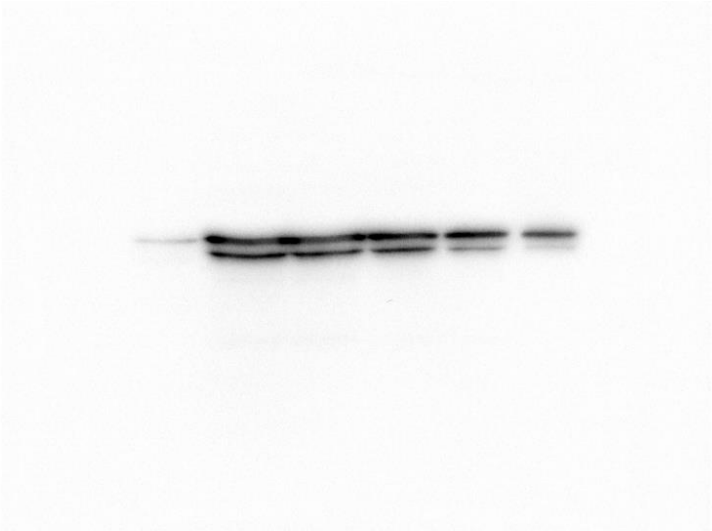

- **α-Tubulin**

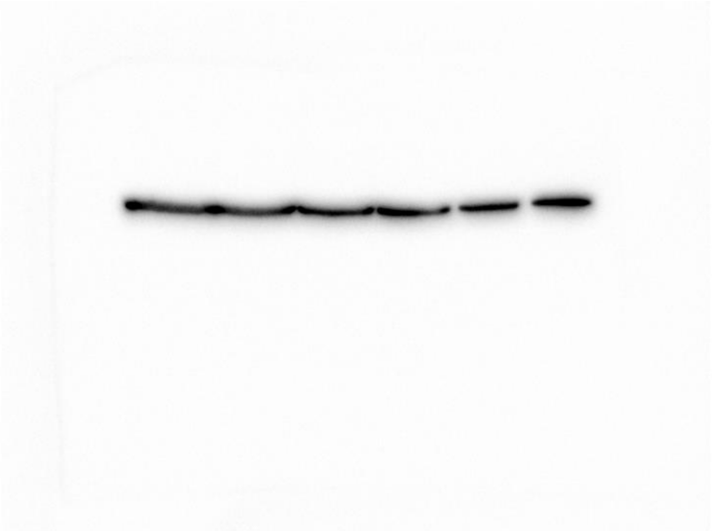

3. **Phosphoplipids**

<u>Replication 1</u>

- **p-NF-κB-p65**

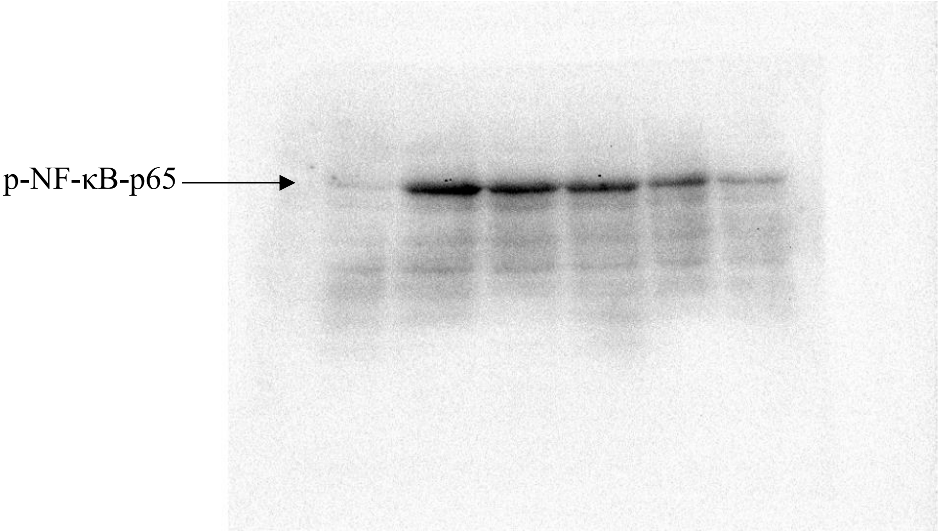

- **p-p38**

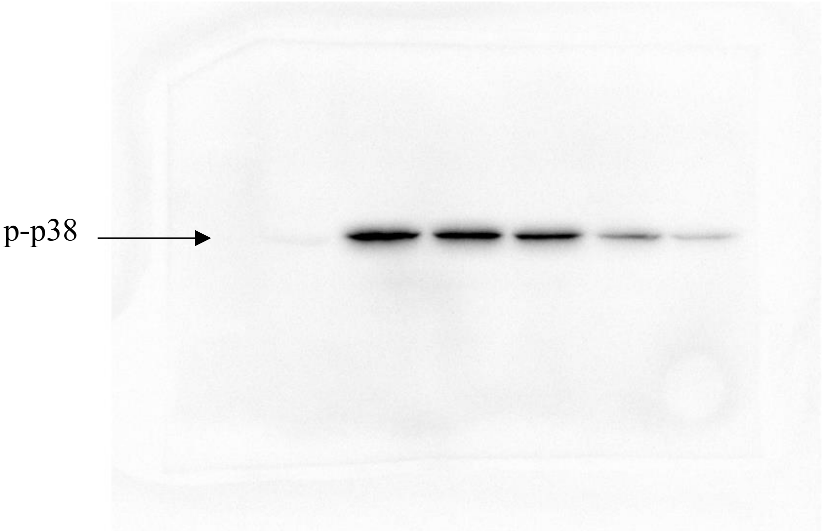

- **p-JNK**

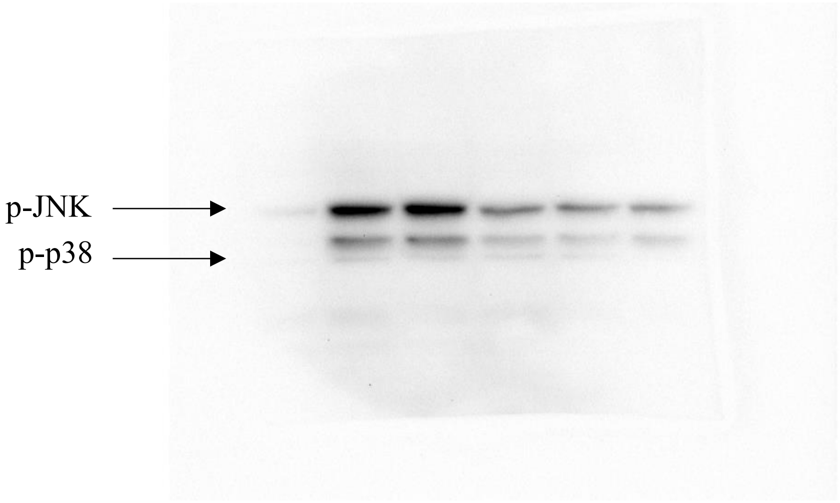

- **p-ERK 1/2 and α-Tubulin**

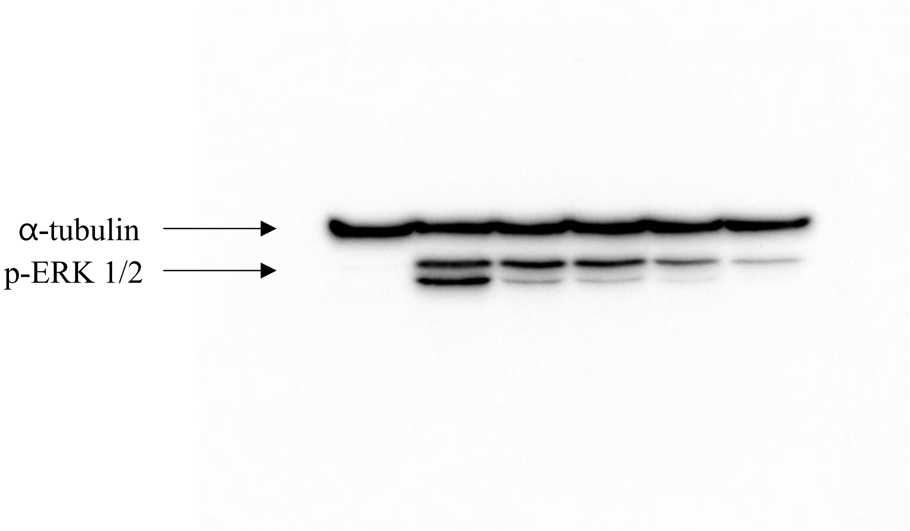

Replication 2

- **p-NF-κB-p65**

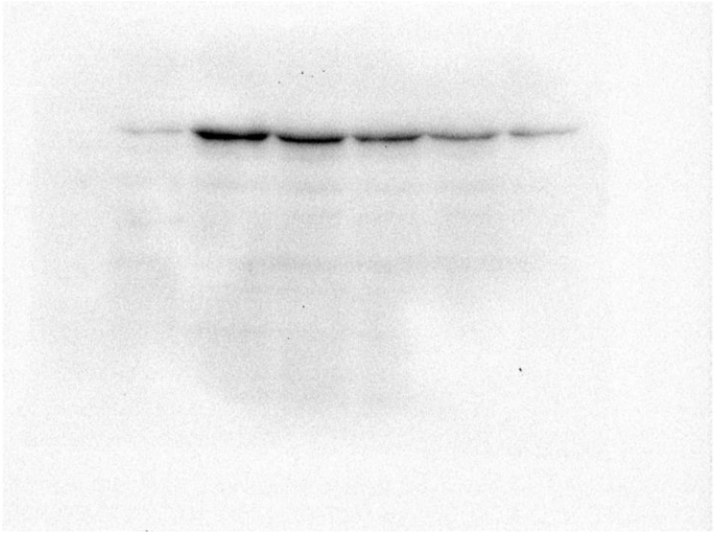

- **p-p38**

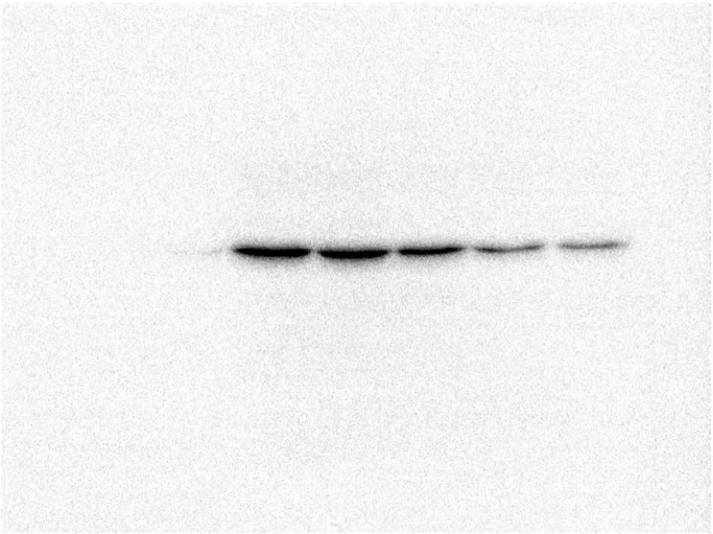

- **p-JNK**

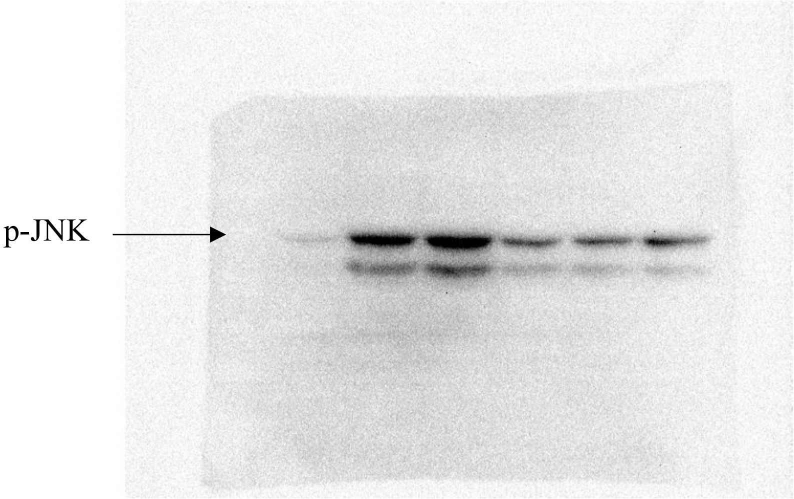

- **p-ERK 1/2 and α-Tubulin**

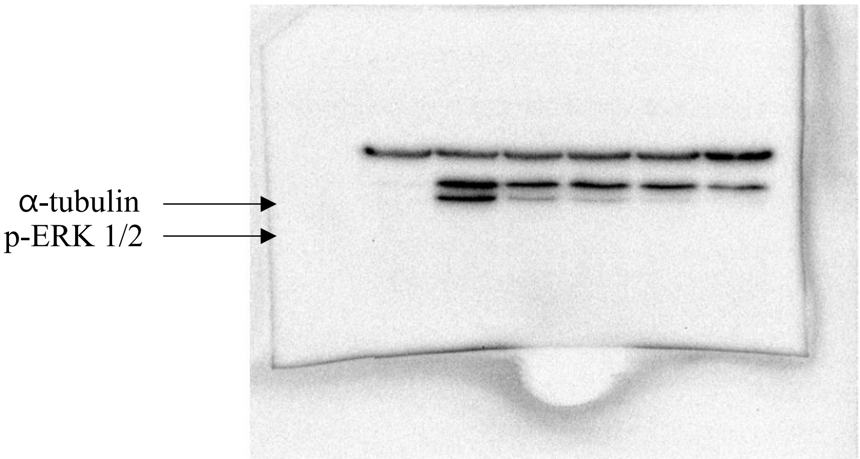

Replication 3

- **p-NF-κB-p65**

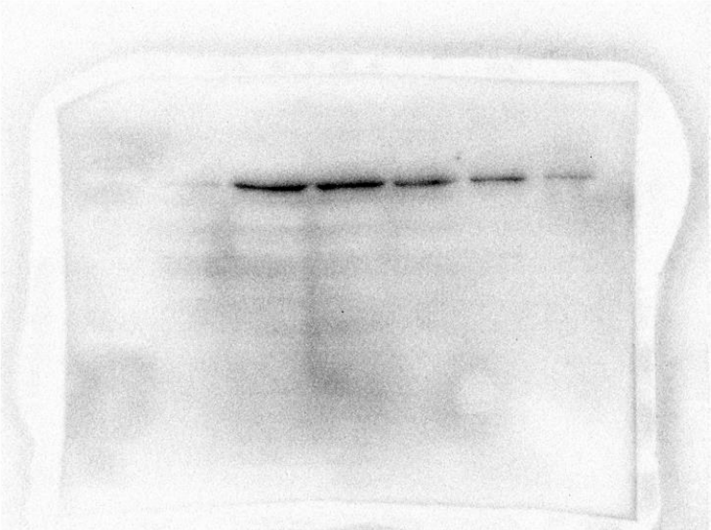

- **p-p38**

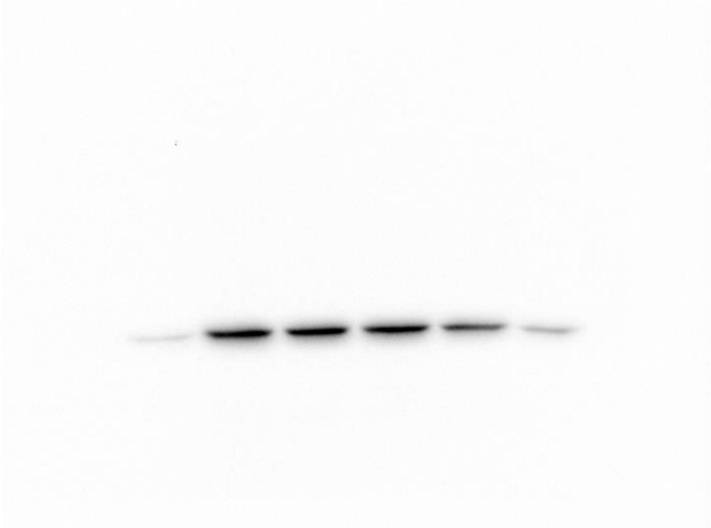

- **p-JNK**

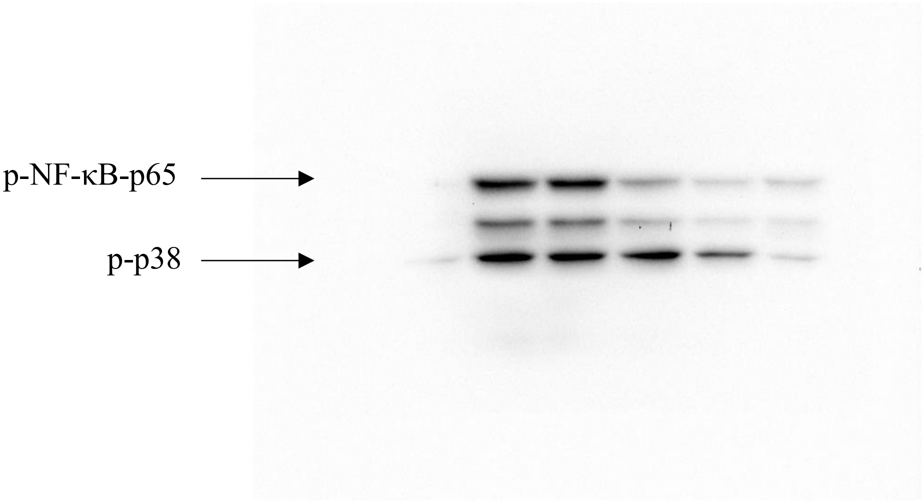

- **p-ERK 1/2**

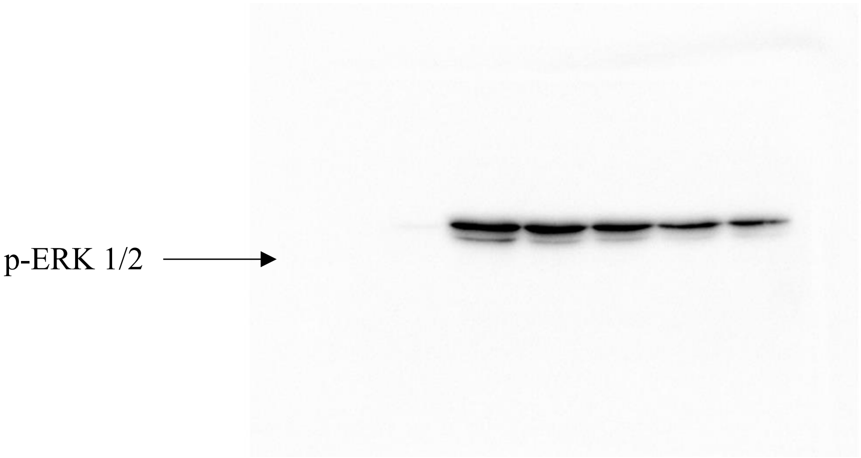

- **α-Tubulin**

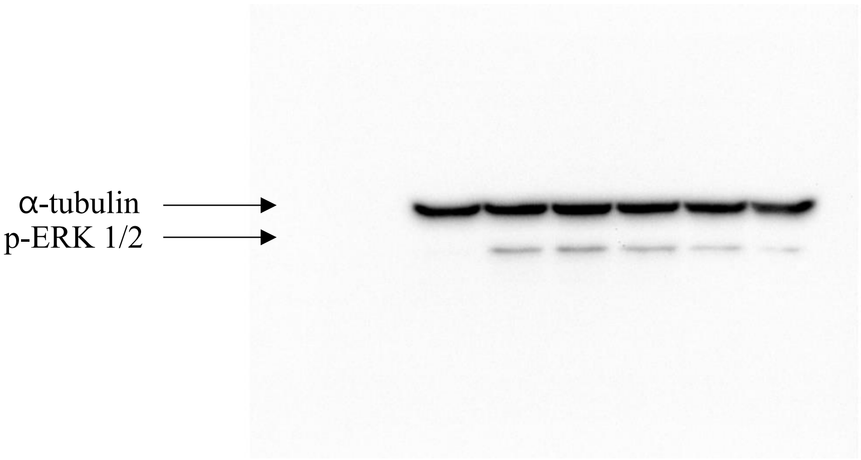

Supplement: S1 Raw images — (PDF) [file pone.0270794.s001.pdf]
