# Supplementary material for: Anti-inflammatory effects of neutral lipids, glycolipids, phospholipids from Halocynthia aurantium tunic by suppressing the activation of NF-κB and MAPKs in LPS-stimulated RAW264.7 macrophages
Source: PLoS One. 2022 Aug 15;17(8):e0270794. doi: 10.1371/journal.pone.0270794 (PMC9377571; doi:10.1371/journal.pone.0270794)
Supplement: S1 Table — (PDF) [file pone.0270794.s002.pdf]

S1 Table. Nucleotide primers used in this study

Gene	Accession No.	Sequence
IL-1β	NM_008361.4	Forward: GGGCCTCAAAGGAAAGAATC<br>Reverse: TACCAGTTGGGGAACTCTGC
IL-6	NM_031168.2	Forward: AGTTGCCTTCTTGGGACTGA<br>Reverse: CAGAATTGCCATTGCACAAC
COX-2	NM_011198.4	Forward: AGAAGGAAATGGCTGCAGAA<br>Reverse: GCTCGGCTTCCAGTATTGAG
TNF-α	D84199.2	Forward: ATGAGCACAGAAAGCATGATC<br>Reverse: TACAGGCTTGTCACTCGAATT
β-actin	NM_007393.5	Forward: CCACAGCTGAGAGGGAAATC<br>Reverse: AAGGAAGGCTGGAAAAGAGC
