# Supplementary material for: Anti-inflammatory effects of neutral lipids, glycolipids, phospholipids from Halocynthia aurantium tunic by suppressing the activation of NF-κB and MAPKs in LPS-stimulated RAW264.7 macrophages
Source: PLoS One. 2022 Aug 15;17(8):e0270794. doi: 10.1371/journal.pone.0270794 (PMC9377571; doi:10.1371/journal.pone.0270794)
Supplement: Attachment — Submitted filename: Response to Reviewers 1.docx [file pone.0270794.s003.docx]

**Reviewer #1**

The manuscript describes anti-inflammatory effects of lipid extracted from *Halocynthia aurantium*. Even though results are interesting, need major revision to enhance the quality of the paper suitable for publication in PLOS ONE.

**Major comments**

1. Author described the extraction method of total lipids (lines 86-87), unfortunately no extraction yield was given anywhere. Similarly, lipid extracted from *H. aurantium* was fractionated into neutral lipid, glycolipid and phospholipids (line 94-96), and neither initial lipid weight nor the fractions yield was given. This is important information to understand the value of the lipid from *H. aurantium.*

- It was corrected in Line 87-88 and 92-95.
- Total lipids produced a yield of 33.6 mg (w/w) or 0.75% of dry material (4.5 g). If 4.5 g of dried material is 100%, yield (33.6 mg) is 0.75% of dried material (4.5 g).
- The neutral lipids, glycolipids, and phospholipids were shown to have a high lipid yield of 8.63 (2.9 mg), 30.95 (10.4 mg), and 44.94 (15.1 mg) % of total lipid (33.6 mg), respectively.
- Thanks.

2. Figures are in poor quality possible due to electronic submission process.

- It was corrected.
- Thanks.

3. Figure 1 described the percentage of individual fatty acids in terms of total fatty acid count. It will not give exact percentage of the fatty acid in the tested fractions i.e., neutral lipid, glycolipids or phospholipids. Lipid fraction such as neutral lipids may contain other components too, better to present the fatty acid data in w/w basis.

- Based on an internal standard, fatty acids were analyzed.
- We are attaching the percentage of fatty acids like the following.
- We calculated all detected lipids by the equation as (each lipid/all fatty acids) * 100.
- Thanks.
- **Table** Fatty acid composition (%) of neutral lipids, glycolipids, and phospholipids from  *H. aurantium* tunic.

| **Fatty Acids** | **Neutral lipids** | **Glycolipids** | **Phospholipids** |
| --- | --- | --- | --- |
| 14:0 | 12.27±1.48^c^ | 1.50±0.63^h^ | 8.51±2.31^c^ |
| 16:0 | 33.98±3.94^a^ | 27.12±0.26^a^ | 26.24±0.85^b^ |
| 18:0 | 17.50±4.71^b^ | 24.08±0.21^b^ | 33.05±2.60^a^ |
| 20:0 | n.d. | n.d. | 1.66±0.22^ef^ |
| Total SFAs | 63.75±2.80 | 52.70±0.89 | 69.45±4.57 |
| 16:1n7 | 4.92±0.58^d^ | 8.16±0.28^d^ | 7.25±3.03^cd^ |
| 18:1n9 (OA) | 11.99±1.34^c^ | 2.96±0.33^fg^ | 6.82±2.57^cd^ |
| 18:1n7 | n.d. | 3.75±0.82^ef^ | 2.44±0.18^ef^ |
| Total MUFAs | 16.92±0.85 | 14.87±1.30 | 16.52±3.56 |
| 18:2n6 (LA) | 13.87±1.80^bc^ | 2.24±0.44^gh^ | 5.43±3.26^ced^ |
| C18:3n3 (ALA) | n.d. | n.d. | 1.39±0.45^f^ |
| 18:4n3 | n.d. | 22.20±0.52^c^ | 3.64±0.73^def^ |
| 20:4n6 (ARA) | n.d. | 3.51±0.11^ef^ | 1.27±0.43^f^ |
| 20:5n3 (EPA) | 5.46±0.63^d^ | 4.49±0.64^e^ | 2.29±0.53^ef^ |
| Total PUFAs | 19.34±1.97 | 32.44±0.96 | 14.03±2.23 |

Results represent means ± SD (*n* = 5). 3), n.d. (not detected)

^a–h^ The letters indicate significant differences (*p <* 0.05) between the amounts of fatty acids, which were obtained from the same fractionated lipids of *H. aurantium* tunic.

4. No positive control was used in any of the bio-assays. Recommended to have positive control for comparison.

- Normally the anti-inflammatory effects used LPS as positive control.
- Thanks.
- References
  - Fernando, I. S., Sanjeewa, K. A., Kim, H. S., Kim, S. Y., Lee, S. H., Lee, W. W., & Jeon, Y. J. (2017). Identification of sterols from the soft coral *Dendronephthya gigantea* and their anti-inflammatory potential. *Environmental Toxicology and Pharmacology*, *55*, 37-43.
  - Li, M., Zhang, L., Cai, R. L., Gao, Y., & Qi, Y. (2012). Lipid‐soluble extracts from *Salvia miltiorrhiza* inhibit production of LPS‐induced inflammatory mediators via NF‐κB modulation in RAW264.7 cells and perform anti-inflammatory effects *in vivo*. *Phytotherapy Research*, *26*(8), 1195-1204.
  - Kim, K. N., Ko, Y. J., Yang, H. M., Ham, Y. M., Roh, S. W., Jeon, Y. J., ... & Oda, T. (2013). Anti-inflammatory effect of essential oil and its constituents from fingered citron (*Citrus medica* L. var. *sarcodactylis*) through blocking JNK, ERK and NF-κB signaling pathways in LPS-activated RAW 264.7 cells. *Food and Chemical Toxicology*, *57*, 126-131.
  - Wang, T., Wu, F., Jin, Z., Zhai, Z., Wang, Y., Tu, B., ... & Tang, T. (2014). Plumbagin inhibits LPS-induced inflammation through the inactivation of the nuclear factor-kappa B and mitogen activated protein kinase signaling pathways in RAW 264.7 cells. *Food and chemical toxicology*, *64*, 177-183.

5. Author described the final concentration of the lipids 20 mg/ml (line 97), but in Figures 2-4 results are described in percentage lipids, need further clarification how the test sample were prepared from 20 mg/ml to 0.5-4% lipids.

- It was corrected in Line 99.
- The stock of lipids has some mistakes in that the final concentration of lipids is 2 mg/mL.
- Stock of lipids has concentration at 2 mg/mL (w/v) as 100%, and then lipids were diluted to 0.5, 1.0, 1.5 and 2.0% (v/v) with medium.
- Thanks.

6. At 4.0% lipid concentration neutral lipid and glycolipids fractions showed cytotoxicity effect (Figure 2A, lines 171-174). No explanation was given why these lipids fractions were tested at 4.0% for NO and other assays. NO inhibition and other positive effect at this concentration may due to cell death.

- Figure 2A showed cell proliferation in RAW264.7 cells (in the absence of LPS) that are toxic at high concentrations while cell proliferation in LPS-stimulated RAW264.7 cells showed non-toxicity.
- The purpose of our study is to test the anti-inflammatory effects. Therefore, we tested the three lipids with the addition of LPS. It showed the highest NO production at a concentration of 4%, especially phospholipids, which reduced NO production by 9.52 % while causing no toxic in the cells.
- We changed the previous one to new figure 2A including the addition of LPS.
  - Figure 2A in the absence of LPS

- LPS condition

- In our study, the three lipids showed the highest NO production at a concentration of 4%, especially phospholipids, which reduced NO production by 9.52 % while causing no toxic in the cells.
- All experiments with three lipids should use the same concentration.
- Following references showed the cell viability of a sample to be lower than control (≤ 80%), and that they can also be evaluated for NO production, gene expression, and protein expression.
- References
  - Qiao, J., Xu, L. H., He, J., Ouyang, D. Y., & He, X. H. (2013). Cucurbitacin E exhibits anti-inflammatory effect in RAW 264.7 cells via suppression of NF-κB nuclear translocation. Inflammation Research, 62(5), 461-469.
  - Konishi, I., Hosokawa, M., Sashima, T., Maoka, T., & Miyashita, K. (2008). Suppressive effects of alloxanthin and diatoxanthin from *Halocynthia roretzi* on LPS-induced expression of pro-inflammatory genes in RAW264. 7 cells. Journal of Oleo Science, 57(3), 181-189.
  - Orecchini, E., Mondanelli, G., Orabona, C., Volpi, C., Adorisio, S., Calvitti, M., ... & Belladonna, M. L. (2021). *Artocarpus tonkinensis* extract inhibits LPS-triggered inflammation markers and suppresses RANKL-induced osteoclastogenesis in RAW264. 7. Frontiers in Pharmacology, 2417.
  - Li, C. Y., Meng, Y. H., Ying, Z. M., Xu, N., Hao, D., Gao, M. Z., ... & Ying, X. X. (2016). Three novel alkaloids from *Portulaca oleracea* L. and their anti-inflammatory effects. Journal of Agricultural and Food chemistry, 64(29), 5837-5844.
  - Yang, H., Xue, Y., Kuang, S., Zhang, M., Chen, J., Liu, L., ... & Deng, C. (2019). Involvement of Orai1 in tunicamycin-induced endothelial dysfunction. The Korean Journal of Physiology & Pharmacology, 23(2), 95-102.
